# Retraction Note: Assessment of fetal intraventricular diastolic fluid dynamics using ultrasound vector flow mapping

**DOI:** 10.1186/s12872-024-03813-2

**Published:** 2024-03-14

**Authors:** Qinglan Shu, Yi Wang, Xinyi Lin, Shenghua Xie, Zhengyang Wang, Sijia Wang, Lixue Yin

**Affiliations:** 1 Ultrasound in Cardiac Electrophysiology and Biomechanics Key Laboratory of Sichuan Province, Sichuan Provincial People’s Hospital, Sichuan Provincial People’s Hospital, University of Electronic Science and Technology of China, Chengdu, China; 2 Department of Cardiovascular Ultrasound & Noninvasive Cardiology, Sichuan Provincial People’s Hospital, University of Electronic Science and Technology of China, Chengdu, China; 3 School of Biomedical Sciences, Faculty of Medicine, Li Ka Shing, The University of Hong Kong, Hong Kong, China

**Retraction Note: BMC Cardiovascular Disorders 23, 488 (2023)**

**https://doi.org/10.1186/s12872-023-03524-0**

The Editor has retracted this article because, owing to an administration error in the Publisher's editorial office, it was published before the peer review process had been undertaken. Post-publication peer review raised concerns that cannot be easily addressed by post-publication corrections as additional work is required. The authors have been invited to submit a new revised manuscript that will undergo robust peer review. The Publisher apologises to the authors for the administrative error. Qinglan Shu disagrees with this retraction. Qinglan Shu has stated on behalf of all the co-authors that they disagree with this retraction.

